# A balanced approach to using organizational patient safety incident data for research

**DOI:** 10.1177/08404704251331179

**Published:** 2025-04-29

**Authors:** Laura D. Pozzobon, Ashley Tattersall, Sarah Tosoni, AnnMarie Edward, Ann Heesters, Carole Garmaise, Michael W. Caesar, Tara Marshman, Lucas B. Chartier

**Affiliations:** 1 7989 University Health Network, Toronto, Ontario, Canada.; 2 7989 University of Toronto, Toronto, Ontario, Canada.; 3 7989 Queen's University, Kinsgton, Ontario, Canada.

## Abstract

Reported patient safety incidents offer high-value perspectives on safety threats but can be an untapped source of learning due to their sensitive nature and the presence of potential data protected under Quality Assurance (QA) legislations. There are no published guidelines for leaders to enable ethical use of data protected under QA legislation in reported patient safety incidents within the Canadian context. Liberating this data requires understanding the appropriate purposes for use, which draws on ethical and privacy-related considerations. We describe the approach followed to balance the duty to protect relevant privacy interests with the moral obligation to conduct research, and the proactive prevention of patient harm at our Canadian multi-site academic health sciences centre. Overall, we developed guidelines and discovered leaders must commit to establishing connections between organizational governance, legal structures, and privacy experts to support research enabling learning from patient safety incidents.

## Introduction

Healthcare professionals work within complex systems where avoidable patient harm is often the result of multiple interconnected factors, and it is almost never the result of healthcare providers’ malicious intent.^1,2^ The Hippocratic Oath is best known for its articulation of physicians’ duty to do no harm, and all healthcare professionals are similarly committed to the moral imperative of non-maleficence. In the context of patient safety, this involves acting to prevent patient harm. Yet, there remains a significant global burden of avoidable patient harm arising from the delivery of healthcare.^3-5^

Healthcare organizations have a moral responsibility to ensure safe care delivery and prevent avoidable patient harm. To identify and mitigate risk factors for harm within systems (e.g., lack of organizational policy, faulty equipment, and gaps within processes), healthcare organizations often implement Incident Reporting and Learning Systems (IRLSs).^6^ Within Canada, IRLSs are a Required Organization Practice for institutions seeking to remain in good standing with Accreditation Canada.^7^ These administrative systems provide a platform for reporting instances of past patient harm or situations with the potential to cause patient harm.^8^ Further, these systems help organizations to capture data on past harm, which constitutes one of the important metrics in measuring and monitoring safety within healthcare.^9,10^ Common data points captured in IRLSs include the type of incident (e.g., medication error), the patient outcome attributed to the incident, patient characteristics (e.g., age), information on the circumstances of the incident (e.g., where the incident occurred and who was involved), contributing factors leading to the incident, organizational outcomes, detection methods (actions leading to incident discovery), mitigating factors, ameliorating actions, and the actions taken to reduce the risk of a similar incident occurring.^11^

For IRLSs to be successful, an organization must have a strong just culture where leaders and healthcare professionals take accountability for the systems they design, and where those who are involved in incidents/incident reporting can trust they will be treated fairly and equitably.^12,13^ Organizations that do not foster a just culture can deter individuals from reporting, thereby making it more challenging to detect and mitigate safety threats within the system.^14^ To promote a culture supportive of learning and improving from incidents, all Canadian provinces have introduced enabling legislation for quality assurance.^15,16^ In general, provincial quality assurance legislation aims to ensure that patient safety incident analyses are done in a transparent and open manner to encourage participation and system improvement.^16^ To support this aim, legislation often prohibits aspects of the information collected for the analysis from being disclosed (e.g., in a legal proceeding). For example, Manitoba’s *The Health System Governance and Accountability Act*^17^ encourages the reporting and investigation of critical patient safety incidents, and aspects of the investigation (e.g., opinions in a prepared document for a critical incident review committee) are considered confidential and privileged under this act to encourage open participation in reviews. Similarly, in Ontario, *the Quality of Care and Information Protection Act*, 2016 (QCIPA) supports open discussions concerning patient safety incidents and prohibits the disclosure of “Quality of Care Information” (QCI) (e.g., discussions, opinions, and deliberations on the patient safety incident by the hospital’s Quality of Care Committee) in legal proceedings.^18,19^ QCIPA also outlines what is *not* QCI and this is described in Box 1. A summary of patient safety incident legislation by province is available from Healthcare Excellence Canada.^16^

## Box 1

*What is not QCI as per QCIPA (2016)*

“‘Quality of care information’ does not include any of the following:

1. simpleInformation contained in a patient record.
2. Information contained in a record that is required by law to be created or to be maintained.
3. Information relating to a patient in respect of a critical incident that describes
  (i.) facts of what occurred with respect to the incident,
  (ii.) what the quality of care committee or health facility has identified, if anything, as the cause or causes of the incident,
  (iii.) the consequences of the critical incident for the patient, as they become known,
  (iv.) the actions taken and recommended to be taken to address the consequences of the critical incident for the patient, including any healthcare or treatment that is advisable,
  (v.) the systemic steps, if any, that a health facility is taking or has taken in order to avoid or reduce the risk of further similar incidents.
4. Information that consists of facts contained in a record of an incident involving the provision of healthcare to a patient.
5. Information that a regulation specifies is not quality of care information and that a quality of care committee collects or prepares after the day on which that regulation comes into force.”

Source: Quality of Care Information Protection Act, 2016 (QCIPA).

In the province of Ontario, where this work was completed, the *Ontario Personal Health Information Protection Act* (PHIPA) describes the conditions under which Personal Health Information (PHI) can be collected, used, and stored for secondary use such as research.^20^ However, QCIPA does not describe the conditions for secondary use of QCI. Leaders working within organizations using IRLSs can be challenged to balance the need to protect the QCI captured from patient safety incident reports under quality assurance legislation, against the duty to use that information to conduct research that can lead to process improvements related to safe and efficient care delivery. We identified an opportunity to provide clarity on how QCI data can appropriately be used for research.

At our large multi-site academic health sciences centre, we sought to develop guidelines to manage the sharing of QCI data stored in our IRLS in a manner that would aim to protect the interests of the public and our organization’s members. The need for guidelines arose from our organization’s intention to use QCI data from reported patient safety incidents to answer research questions with the ultimate goal of improving care delivery. We were challenged by our competing duties to improve care delivery and prevent harm for future patients whilst doing no harm to our employees, organization, or just culture by using reported patient safety incident data. To the best of our knowledge, there is no published guidance for leaders that outlines how to enable the ethical use of QCI from patient safety incident reports and reviews found in IRLSs for research involving a single organization within the Canadian context. The lack of such guidance inhibits individual organizational and system-wide learning that can prevent future patient incidents. We regarded this gap as an ethical and pragmatic shortcoming that ought to be addressed.

The purpose of our article is to describe the process we took to enable the use of QCI in our IRLS for research in a manner that balances respect for confidentiality with the ethical obligation to improve care and prevent patient harm. Further, we provide the resulting guidance on how to use QCI collected in our organization’s IRLS for research purposes to support other organizations in their research efforts to improve patient safety. Liberating the use of this data required navigation of the policy landscape, legislation, organizational governance, purpose of use, as well as ethical and privacy-related considerations.

## The ethical challenge

There is widespread recognition of the importance of learning from past instances of patient harm to make improvements in healthcare delivery.^11^ An ethical issue arose for us when exploring a potential research study in the domain of patient safety. Specifically, we asked ourselves, “should QCI collected in our IRLS be used for research?” The steps required to use PHI collected in our IRLS for research was clear, as the Ontario PHIPA describes the conditions under which PHI can be collected, used, and stored for secondary use. However, as QCIPA does not expressly address secondary use of QCI, how to use this data for research purposes was unclear. Historically, QCI was used solely for the purpose of organizational process improvement and not for research purposes at our organization.

Deliberations on whether the QCI data in the IRLS should be used for research surfaced tensions between competing ethical principles, specifically the principles of non-maleficence and the duty to be trustworthy. In the context of research, non-maleficence is implicated with efforts to avoid or minimize harms to those directly or indirectly involved in research procedures or through the primary or secondary use of their data.^21^ As a general principle, research should not violate an individual’s autonomy even if the research is suspected to have great value. Trust in individuals or institutions can be undermined when expectations are violated, such as when patients’ or a care provider’s thoughts/opinions/deliberations related to patient safety incidents is used in ways not consistent with their understandings of the purposes to which they had consented. Non-maleficence and trust are also entrenched within the domain of patient safety. Ultimately, patient safety is, “a framework of organized activities that creates cultures, processes, procedures, behaviors, technologies and environments in healthcare that consistently and sustainably lower risks, reduce the occurrence of avoidable harm*,* make errors less likely and reduce impact of harm when it does occur”.^3^ Further, in order to achieve reductions in preventable harm, trust (as seen in a just culture) from those engaging in the reporting and review process of reported harm is required.^13^ Specifically, trust that organizations will make improvements from reported incidents, and protect the confidentiality of QCI gathered through reporting and review processes, is paramount in engaging individuals in IRLSs.

We recognized that using the QCI collected in our IRLS for research without appropriate safeguards had potential to indirectly put individuals at increased risk for harm (e.g., via breach of confidentiality) and/or damage their trust in the organization. Further, published results of research studies using this data could lead the public to make worrying inferences about the overall safety of the organization and potentially have an impact on the public’s sense of the organization’s trustworthiness.

## A balanced approach to using patient safety incident data

### Organizational setting

This work was conducted at a multi-site academic hospital in Ontario, Canada. This organization implemented a robust IRLS process in 2015 to support movement towards becoming a high reliability organization.^22^ Incidents are reported anonymously in an electronic reporting system by employees, physicians, and volunteers (i.e., those volunteers hired by the organization) within the organization. The review of potential reported critical incidents is done under QCIPA and is led by a central Quality & Safety department in conjunction with other healthcare professionals and leaders on behalf of the organization’s quality governance body (Quality of Care Committee). Findings from reviews, including potential QCI (e.g., contributing factors and mitigations), are also documented in the electronic reporting system on the incident file. Overall, data is captured in our IRLS in alignment with all of the ten patient safety concepts identified in the WHO’s International Classification for Patient Safety (ICPS).^11^

### Method

**Figure 1.**
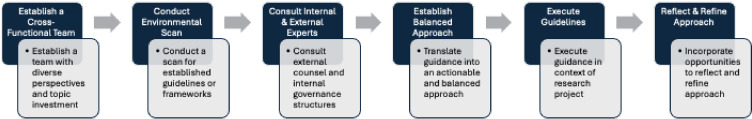
Method to establish a balanced approach using patient safety incident data from an incident reporting and learning system.

Our decision to develop a balanced approach to using patient safety incident data from our IRLS for a research study was supported by creating a cross-functional team allowing us to navigate socio-political environments and to learn from experts in privacy, law, and ethics. The team contemplated the ethical challenge and identified two main actions that could be taken: (1) do not proceed with the research study or (2) develop guidance to support the research study with safeguards to mitigate risk with the use of QCI. The approach taken by the team to enable us to develop a balanced approach is summarized in Figure 1.

Overall, a team comprised of leaders representing different functions within the organization (quality and safety; privacy and risk; clinical and organizational ethics; and data and analytics) was struck to consider the potential risks and benefits of using QCI from patient safety incident reporting and reviews in our organization’s IRLS. The team conducted an informal environmental scan to identify published guidelines enabling the ethical use of patient safety incident data collected under quality assurance legislation within the Canadian context. While collective support for such a use was found, no guidelines or established frameworks were identified specific to enabling the use of IRLS at an individual institution for research was found. Although the National System for Incident Reporting Privacy Impact Assessment (NSIR-PIA) was explored and offers considerations specific to privacy of the NSIR data, the document does not explicitly consider QCI data or outline considerations for secondary use of the data for research. In the absence of established guidance, external counsel was engaged to assess risks and formulate appropriate safeguards. Steps were also taken to seek guidance and support from key organizational governance structures, including committees and executive groups with the responsibility to protect QCI and improve patient care.

On balance, the team believed that developing guidance to support research with safeguards to mitigate risk was more justifiable than failing to embrace that opportunity. We reasoned that there is potential to maximize the value of QCI collected in IRLS through research rather than by limiting its impact by restricting our insights to more local quality improvement projects. Specifically, we understood that insights derived from our data can support a wider group of researchers interested in advancing the field of patient safety thereby leading more broadly to reductions in preventable patient harm. Authors of the WHO’s Global Patient Safety Action Plan have also acknowledged that further research is needed to generate knowledge to reduce preventable patient harm.^3^ Furthermore, we noted that data collected (e.g., contributing factors) from IRLSs are already being used in other countries for research purposes.^23^ In addition, using data collected in our organization’s IRLS for research *with appropriate safeguards* aligns with our organization’s overarching purpose which is to transform lives and communities through excellence in care, discovery, and learning. Finally, an effort to liberate data and share the benefits of that effort aligns with our organization’s aim to deliver the safest care possible and contribute in a positive way through teamwork and collaboration. Guidance from many stakeholders was translated into an actionable approach used to navigate a complex socio-political environment. Incorporating opportunities to reflect throughout was critical to refining the approach based on evolving perspectives and newly uncovered information. As we move forward with these guidelines, we will continue to iterate as needed. With each research study done with QCI data, there is an opportunity to collect feedback from researchers using the guidelines to make improvements.

## Results

The cross-functional team led the work of aligning existing organizational structures to develop a balanced approach to the use of patient safety incident data, including QCI (extracted from the organization’s IRLS), for research purposes. The resulting guidance can be found in Table 1.

**Table 1.** Guidance on how to enable the ethical use of reported patient safety incident data containing QCI for research.

Guidance	Rationale
Proposals for research studies using data captured in the organization’s IRLS (QCI and PHI) must be reviewed and approved by the data stewards of the system	The steward of the data collected in the organization’s IRLS is the Quality of Care Committee (QCC) with oversight from the Quality of Care Committee Executive (QCC Exec). Data stewards have a responsibility for managing the data (e.g., collection, storage, integrity, and security) and have a responsibility to respect the interests of those who may be affected by how the data is managed and used.^24^ The QCC is composed of executive leaders representing various functions across the organization as well as patient partners. It was determined that approval and endorsement of research proposals using data from the IRLS would need the data steward’s approval. This function was also added to the QCC’s Terms of Reference and aligns with the organization’s data stewardship policy.
Researchers must develop a proficiency in patient safety data literacy	Historically, at our organization, QCI was used solely for process improvement. Such use can result in a knowledge gap for those who do not regularly interact with and use QCI. Education is fundamental to ensure all involved in the research are aware of the complexities of patient safety data, thereby creating a common understanding on the basis of which conversations can move forward.
Researchers must connect with leaders of the organization’s Quality & Safety team to understand the data captured in the organization’s IRLS	The organization’s central Quality & Safety team has the expertise to understand and interpret the data captured in the IRLS. By meeting with this team, the researchers can understand better what data is available and determine the minimal use of data fields required to answer their research question(s). Researchers are encouraged to access the most minimal dataset necessary. There is also an opportunity to add the central Quality & Safety team as a reviewer in the organization’s Research Ethics Board (REB) process.
The organization’s Privacy and Legal team must review the data fields proposed to be used in the study	Some fields within the IRLS may contain QCI that must be protected from disclosure under the provincial legislation governing the review of patient safety incidents. It was determined that the Privacy and Legal teams must review the fields research teams deem necessary to answer their research question(s) and to ensure that QCI is not disclosed inadvertently. The Privacy and Legal teams at our organization have the expertise to determine which data fields are considered QCI and can consult external counsel as needed.
A centralized process for internal requests for data from the organization’s IRLS should exist	A centralized process administratively managed by the central Quality & Safety team (rather than the organization’s Decision Support team) enables incorporation of key expertise and knowledge into requests for data from the organization’s IRLS and acts as a safeguard to ensure best practices and guidelines are followed. Data requests are received, reviewed, and completed by the central Quality & Safety team through a standardized request form. At the time of request, the requester agrees to certain terms of use based on their purpose (quality assurance, quality improvement, or research). This was a pre-existing process prior to this ethical challenge being contemplated but has been identified as a key process to prevent inappropriate use of QCI.
Dissemination must be reviewed by the organization’s Privacy and Legal team	Any dissemination of products from a research study must first be reviewed by the Privacy and Legal team (e.g., abstract submissions for conferences, manuscripts, and presentations). Although this currently is a labour-intensive process, it is an important step in ensuring the data shared is properly de-identified and aggregated to protect the anonymity of those who experienced patient safety incident/were involved in a patient safety incident, and to prevent inadvertent disclosure of QCI. This review is strictly focused on privacy and is not focused on changing content or opinions/recommendations proposed by the authors.

The guidelines provided in Table 1 are specific to the secondary use of patient safety incident reports, including QCI, for research purposes. The cross-functional team also acknowledged that the routine Research Ethics Board review process is required for research involving the use of PHI and QCI to ensure alignment with the Tri-Council Policy Statement (TCPS2) and any other relevant normative standards (e.g., Canadian Institute for Health Research’s Best Practices for Protecting Privacy in Health Research).^25,26^ In Table 2, we identified a list of specific and routine research ethics practices that are integral to research using QCI. We believe our guidelines (in Table 1) complement and augment customary ethics safeguards by further mitigating risk for inadvertent disclosure of QCI.

**Table 2.** Routine research ethics practices integral to safeguarding reported patient safety incident data containing PHI and QCI.

Guidance	Rationale
Data users must be affiliated with the organization	Users of the data from the organization’s IRLS must be affiliated with the organization (e.g., as employees). As part of their onboarding to the organization and continued affiliation, these data users receive training on the organization’s privacy policies and practices (e.g., annual privacy training). This affiliation also ensures they adhere to and are bound by privacy, access and use policies.
The research study must be reviewed by the organization’s REB	To ensure the research study meets ethical standards and is attentive to risks to patients and the institution, the research proposal must be reviewed and approved by the organization’s Research Ethics Board (REB) rather than the organization’s Quality Improvement Review Committee. REBs and researchers are expected to minimize privacy risks associated with secondary data use in alignment with the Tri-Council Policy Statement: Ethical Conduct for Research Involving Humans—TCPS 2 (2022).^25^ REB approval is required before any data can be released to the data users.
If external parties will be involved in data use, a Data Sharing Agreement (DSA) is required	If external parties are involved in a research study, execution of the DSA is required before any data can be released to the data users. A DSA between organizations that would have access to the data describes who will have access to the data, and how it can be used, stored, and destroyed. This is required to prevent misuse of the data. Additional terms have been incorporated into the DSA to acknowledge the heightened confidentiality of QCI and to affirm that the data and QCI will be used exclusively for the specified research purposes. The DSA is granted exclusively to a designated individual, rather than an organization. Any requests for access by additional external users will be evaluated on a case-by-case basis.
Only after REB approval is granted and the data sharing agreements (if required) are executed, can data from the IRLS be requested and released from the central Quality & Safety team	Data will only be released from the central Quality & Safety team to the appropriate data users once it is determined that the study meets ethical standards and safeguards have been put in place to prevent misuse of the data. As part of the request for data from the organization’s IRLS, requesters must indicate the intended use in the standardized form. If research is the selected purpose, the REB approval number must be provided. Further, the requester agrees to the documented terms of use.
Data used from the IRLS must be de-identified	Data provided to the appropriate data user will be identified in alignment with the organization’s policy. For instance, if knowing the age of patients who experienced a patient safety incident is required, the age or age range will be provided instead of the date of birth captured in the IRLS. This is to protect patient privacy. Strategies to suppress low volume data (e.g., number of safety incidents per clinical unit) should also be employed to protect patient privacy.

## Leadership lessons learned

Leaders within healthcare organizations are responsible for creating clarity for teams when ambiguity threatens to undermine safety and transparency. The members of our cross-functional team developed guidance on enabling the ethical use of QCI in reported patient safety incidents for research where no guidance previously existed. Through navigating the development of these guidelines, we saw the value of engaging diverse perspectives to reach a common goal, build coalitions, and increase patient safety data literacy.

When our cross-functional team was developed, representatives from multiple organizational functions were selected to ensure diverse perspectives were considered when contemplating the ethical risks, opportunities, and potential actions the organization could take. Each representative contributed expertise which supported a comprehensive view of the potential impact the decision might have on individuals whose data is stored in our IRLS, and on perceptions related to institutional trustworthiness. In addition to the need to engage diverse perspectives, it became evident that the guidance we developed should leverage existing governance and organizational processes. As our organization is a multi-site academic healthcare centre, we were well-situated to build upon existing structures. Specifically, we purposefully connected our guidelines with our organization’s existing quality governance structure, research ethics review process, policies, and internal data request procedures for accessing IRLS data. It was through the engagement of these various constituencies and bodies that we learned of the structures that we were able to leverage in order to develop the guidance.

Finally, as a result of this process, it became evident there was poor patient safety data literacy outside of the organization’s central Quality & Safety team. In order to understand the data that we contemplated sharing, just-in-time education on QCIPA and the patient safety information captured in the organization’s IRLS was required for the cross-functional team and researchers.

We recognize that our guidelines may not be directly transferable to other organizations and, thus, we encourage health leaders to consult experts in the legislation governing patient safety practices in their own jurisdictions. By engaging with experts and providing learning opportunities for others, leaders can enhance learning and development, mobilize evidence to inform practices, and build effective teams that can achieve results with greater impact.^27^

## Conclusion

In conclusion, we have described the process undertaken to develop guidelines to liberate the use of QCI captured in IRLS for research purposes. By developing a cross-functional team with expertise in quality and safety, relevant provincial legislation, organizational data structures, and research ethics, we were able to engage thoughtfully in the exercise of weighing the ethical risks and benefits related to using our organization’s IRLS data for research. Further, this team supported the development of guidelines which articulated our moral obligation to protect those who experienced patient safety incidents (e.g., patients) or were involved in incidents (e.g., healthcare providers), in order to make improvements to prevent further patient harm. We urge other health leaders to consider leveraging their own organizations’ data to enhance patient safety by creating robust processes by accessing diverse perspectives in the service of this common goal. This will require building coalitions, increasing patient safety data literacy, and foregrounding the ethical risks and opportunities associated with this important work. First steps may require substantial ground clearing but the path is worth taking. Leaders may also want to consider sharing the guidelines and adaptations they make over time to support others in their journey of using data from IRLS for research purposes. Moreover, health leaders may consider partnering with researchers to understand healthcare providers’ perspectives on the use of IRLS data for research purposes.
